# Protein rheostasis: quality control in metastable proteomes

**DOI:** 10.1098/rstb.2025.0279

**Published:** 2026-10-01

**Authors:** Michele Vendruscolo

**Affiliations:** Department of Chemistry, University of Cambridge, Cambridge, UK

**Keywords:** protein homeostasis, protein misfolding, protein aggregation, liquid–liquid phase separation

## Abstract

Protein homeostasis is often described as the capacity of cellular quality-control systems to maintain proteome function by favouring functional protein states. Yet many proteins can populate multiple states, including native conformations, liquid-like condensed assemblies, and aggregated states, reflecting the metastability of the proteome. As a framework for understanding how cells preserve proteome function under such conditions, we discuss protein rheostasis as the system that regulates thermodynamic driving forces and kinetic barriers to control the flux between alternative states over time. Framing proteome maintenance in terms of rheostatic control over the multiple states helps rationalize how ageing, stress, and mutations redistribute populations towards condensed and aggregated states by eroding kinetic buffering capacity, and it suggests therapeutic opportunities that restore control by tuning the transitions between metastable states.

This article is part of the Theo Murphy meeting issue ‘ProteostaSys: a systems view of proteostasis’.

## 1. Introduction

Protein homeostasis, or proteostasis, can be defined as the ensemble of cellular processes that regulate the conformations, interactions, concentrations and localizations of proteins so as to sustain a functional proteome [1–5]. In homeostatic control, error detectors and effectors act in feedback loops to keep conditions, such as temperature and pH, within relatively narrow ranges around a fixed set-point. Within this view, native protein states are considered as stable endpoints, and any deviations from these states, such as misfolding or aggregation, result from failures of cellular quality control.

However, emerging evidence regarding protein phase separation and metastability suggests that the maintenance of native states is only one aspect of a broader quality-control system [6–13]. Because a significant portion of the proteome is expressed near its solubility or supersaturation limit, these native states are frequently metastable, and proteins exhibit a rich phase behaviour [14–16].

It is therefore useful to ask how protein homeostasis should be formulated for proteins operating in metastable regimes in which several alternative states are accessible, and in which regulation acts by shaping the fluxes, and hence the steady-state occupancies, between these states rather than by enforcing a single endpoint. In such circumstances, the relevant control variable is the maintenance of a functional distribution of conformations and assemblies through the modulation of both thermodynamic driving forces and kinetic barriers. We refer to this broader mode of regulation as protein rheostasis [17], to emphasize that cellular mechanisms can tune the occupancies and interconversion rates between native, condensed and aggregated states as conditions change, thereby sustaining proteome function within an intrinsically metastable landscape.

## 2. From homeostasis to rheostasis

In this broader picture, protein homeostasis can be seen as a limiting regime that applies when the native state is thermodynamically rather stable and when substantial kinetic barriers suppress conversion into alternative states. Many proteins, however, operate in a metastable domain in which native conformations, liquid-like condensed assemblies and aggregated species are all, in principle, accessible, and the relevant question becomes how cells regulate the flux between these competing basins [14–16].

Protein rheostasis captures this situation by focusing on the dynamic redistribution of protein populations across states and on the mechanisms that tune both the thermodynamic driving forces for self-association and the kinetic barriers that determine the rates of interconversion. In such a regime, cellular control reshapes the free-energy landscape through chaperone action, degradation and disaggregation pathways, post-translational modifications and compartmentalization. Ageing, stress, and disease-associated mutations can then be understood as perturbations that erode this kinetic buffering capacity and bias steady-state populations towards condensed and aggregated states, even when the underlying homeostatic machinery remains present.

## 3. Dynamic regulation at the molecular and cellular levels

Dynamic regulation in biology is increasingly understood to arise from the transitions between biochemical states through shifts in conformational equilibria, activities and interaction patterns [6–13]. Accordingly, the principles of rheostatic control are most clearly seen in a range of representative systems, which illustrate how regulated changes in cellular conditions redistribute protein populations between alternative functional and dysfunctional states.

### (a) Conformational ensembles as rheostatic systems

Disordered proteins provide an example of rheostatic control at the single-protein level. The nuclear co-activator–binding domain (NCBD) exists not as a single structure but as an ensemble of interconverting conformations whose relative populations depend on binding partners, post-translational modifications, and environmental conditions [18]. Rather than stabilizing one preferred conformation, the cellular environment modulates the free-energy differences and interconversion rates between multiple conformational basins, thereby tuning functional output. In this sense, NCBD exemplifies protein rheostasis at the level of conformational states [19]. Function is not maintained through a well-defined unique structure, but by regulating the population distribution across an ensemble of metastable states. More broadly, allosteric proteins can adjust their state in response to thermodynamic or kinetic inputs, often operating by shifting ensemble populations in response to ligands [20,21].

### (b) Proteoforms, networks, and the structure–function continuum as rheostatic principles

Protein rheostasis can be understood through the emerging view that protein function arises from a structure–function continuum rather than from a single, context-independent native state [10,13]. In this continuum, a gene encoding a protein can generate multiple proteoforms, whose types, populations and behaviour depend on cellular conditions, alternative splicing, post-translational modifications and interaction partners [22–24]. From this perspective, the cell can be viewed as a network of networks, in which conformational ensembles, interaction maps and spatial organization are dynamically coupled. Function then emerges not from one fixed structure but from the regulated redistribution of proteins across alternative states, whose occupancies are tuned by rheostatic mechanisms. This view parallels the concept that sequence and context jointly encode functional outcomes, where context-dependent structural ensembles rather than unique folds mediate biological activity.

### (c) Phase separation as a rheostatic mechanism

Liquid–liquid phase separation provides another mechanism of rheostatic regulation. For many proteins, especially those expressed near their solubility limits, the condensed phase represents a metastable basin rather than a terminal endpoint [25,26]. Protein rheostasis in this context involves the regulation of both the thermodynamic stability of the condensed basin (e.g. whether phase separation occurs at all) and the kinetic barriers separating droplets from irreversible aggregation. Cells can tolerate transient occupancy of condensed states and, in some contexts, may use such transitions to regulate function [6,7,9–13].

### (d) Compartmentalization as control of local concentration and basin occupancy

Spatial organization provides a further layer of rheostatic control by regulating local protein concentrations, a key determinant of which states are accessible. Compartmentalization into organelles, membranes, or biomolecular condensates can drive proteins across phase boundaries without altering their intrinsic properties [6–8]. Conversely, sequestration or active transport can reduce local supersaturation and stabilize native states [27,28]. From this perspective, aspects of cellular architecture may contribute to rheostatic regulation by constraining diffusion, local concentration, and interaction networks. Protein quality control thus emerges not only from molecular interactions but from the dynamic regulation of spatial context, which determines whether proteins preferentially occupy native, condensed, or aggregated basins.

### (e) Molecular chaperone buffering as control of kinetic barriers between states

Molecular chaperones act as rheostatic elements by selectively reshaping the kinetic landscape of transitions between different protein states. Rather than simply refolding misfolded proteins, molecular chaperones raise kinetic barriers to aggregation, lower barriers for productive folding, and accelerate clearance pathways, thereby controlling fluxes between native, condensed, and aggregated basins [1,3,4]. Importantly, molecular chaperones do not necessarily abolish alternative states in the protein free-energy landscape, but can bias fluxes between them by reshaping kinetic barriers and effective stabilities. Ageing, stress, or mutations that reduce chaperone capacity effectively weaken this kinetic buffering, allowing increased occupancy of aggregated states without any necessary change in underlying thermodynamic driving forces [1,3,4,29,30]. From the rheostatic perspective, protein homeostasis failure reflects a loss of kinetic control in an inherently multi-stable system.

### (f) Post-translational modifications as rheostatic shifts of phase boundaries

Post-translational modifications provide a means of modulating protein states by altering interaction strengths, solubility limits, and phase boundaries [24]. Phosphorylation, acetylation, or ubiquitination can shift the relative stability of native and condensed states, suppress or promote phase separation, or bias the system away from aggregation-prone regimes [31,32]. In rheostatic terms, post-translational modifications act as continuous tuning parameters that reshape the protein free-energy landscape rather than binary on/off switches. By dynamically adjusting post-translational modification patterns, cells can reposition proteins within multi-stable landscapes, redistributing occupancy among coexisting basins in response to changing physiological demands.

## 4. Implications for disease and therapeutic intervention

This rheostatic perspective suggests that neurodegenerative and systemic protein misfolding diseases may reflect failures in the regulated tuning of the distributions of different protein states. In a metastable proteome, native, condensed, and aggregated states can all be accessible, and pathology emerges when the cell can no longer maintain the dynamic steady-state that limits flux into long-lived, self-templating or clearance-resistant aggregates [9,33,34]. Ageing, chronic stress, inflammation, and disease-linked mutations can not only impair protein synthesis or degradation, but also progressively erode the kinetic buffering capacity that normally keep aggregation slow and reversible and allowing a gradual drift in basin occupancy towards pathological states.

This perspective connects naturally to the long-standing concept of adaptive proteostasis [1,2,35]. In that view, the proteostasis network is not a fixed maintenance module but a tunable control system whose operating point can be shifted pharmacologically. As proteins can populate multiple states, including native, condensed and aggregated forms [34], rheostasis concerns the regulation of their steady-state populations and the rates of interconversion among them, particularly when thermodynamics or kinetics favour self-association.

Therapeutically, this implies that effective intervention should aim to restore rheostatic control by acting on both thermodynamics and kinetics in a context- and compartment-specific manner. For example, small molecules and post-translational modification programmes can reduce supersaturation, or shift phase boundaries so that condensed states remain functional and reversible rather than hardening into aggregation-prone material [36–41]. In practice, this includes strategies that stabilize native conformations, reduce effective concentration through sequestration and transport, or tune interaction valency to maintain liquid-like condensate behaviour [36–41].

Furthermore, cellular protein quality control can itself be viewed as a set of rheostatic elements, in which molecular chaperones, disaggregation machineries, and degradation pathways act primarily by reshaping the kinetics of nucleation, growth and clearance [1,2,33–35,40]. By increasing the barriers to pathological self-association while accelerating reversal and removal, these systems limit flux into long-lived, self-templating aggregates and thereby stabilize the steady-state distribution of protein conformations and assemblies. Therapeutic strategies that enhance proteasomal and autophagic flux, strengthen disaggregation and refolding capacity, and improve trafficking and compartment-specific quality control may therefore restore regulation not by enforcing a single endpoint, but by re-establishing kinetic buffering within an intrinsically multi-stable landscape [1,2,42,43,44].

Building on early proposals to pharmacologically adapt proteostasis [1,2,35], strategies can be seen as attempts to reprogramme the network-level set point and gain across stress-response pathways (heat shock response, endoplasmic reticulum unfolded protein response, integrated stress response), while minimizing chronic maladaptation. In rheostatic terms, these interventions aim to re-establish a regime in which condensed states can be used productively, and aggregates remain both slow to form and fast to clear.

A practical implication is that biomarkers and endpoints should shift accordingly. Rather than focusing solely on the abundance of a single aggregated species, it becomes important to quantify supersaturation and proximity to phase boundaries, material properties and turnover of condensates, and clearance and refolding fluxes that together define the rheostatic operating point. Therapeutic benefit may therefore involve a redistribution of populations across states in the free-energy landscape, achieved by altering kinetic barriers, thermodynamic biases or both.

## 5. Conclusions

Protein rheostasis generalizes the notion of protein homeostasis to the intrinsically metastable regime in which proteins operate across multiple conditions and can access several competing states. Framed in thermodynamic and kinetic terms, it recognizes both the prevalence of phase transitions and the fact that cellular regulation acts continuously to reshape the balance between alternative conformations and assemblies. In this view, protein homeostasis remains a central organizing principle, but it emerges as a limiting case of a broader rheostatic control when the landscape is effectively stable. Whereas adaptive proteostasis describes the regulated deployment of quality-control machinery to adjust folding, trafficking and clearance capacity, rheostasis describes how such regulation reshapes the free-energy landscape to control the populations of multiple protein states and the rates of interconversion among them.

Proteostasis components can be regarded as the dedicated machineries that manage protein fate by controlling synthesis, folding, trafficking, and clearance, thereby buffering the proteome against error and stress [1–5]. Rheostasis instead concerns a broader class of influences, molecular as well as physicochemical, that reshape the distribution of accessible protein states, whether or not their primary biological role is quality control. In this sense, changes in crowding, metabolite and nucleic acid levels, post-translational modification patterns, pH, ionic strength, redox balance, and interaction networks can all bias the populations of native, condensed and aggregated basins, and modulate the kinetic barriers that govern flux between them.

Biological regulation can be viewed through the lens of protein rheostasis as a flow between distinct steady states, each stable against modest perturbations yet capable of being repositioned as conditions change. Living systems therefore preserve proteome integrity by allowing regulated shifts in the preferred distributions of protein states across different operating regimes. By tuning relative stabilities and reshaping metastable equilibria, cells achieve resilience through a controlled flexibility in conformational and assembly populations.

## Data Availability

This article has no additional data.

## Theme

One contribution of 20 to a Theo Murphy meeting issue ‘ProteostaSys: a systems view of proteostasis’
